# Training and assessing convolutional neural network performance in automatic vascular segmentation using Ga-68 DOTATATE PET/CT

**DOI:** 10.1007/s10554-024-03171-2

**Published:** 2024-07-05

**Authors:** R. Parry, K. Wright, J. W. Bellinge, M. A. Ebert, P. Rowshanfarzad, R. J. Francis, C. J. Schultz

**Affiliations:** 1 School of Medicine, The University of Western Australia, Perth, Australia; 2 Department of Cardiology, Royal Perth Hospital, Perth, Australia; 3 School of Physics, Mathematics and Computing, The University of Western Australia, Crawley, WA Australia; 4 Department of Radiation Oncology, Sir Charles Gairdner Hospital, Perth, Australia; 5 School of Medicine and Population Health, University of Wisconsin, Madison, WI USA; 6 Department of Nuclear Medicine, Sir Charles Gairdner Hospital, Perth, Australia

**Keywords:** Gallium-68 DOTATATE positron emission tomography, Cardiovascular inflammation, Coronary artery disease, Artificial intelligence, Deep learning, Neural network, Automatic segmentation

## Abstract

To evaluate a convolutional neural network’s performance (nnU-Net) in the assessment of vascular contours, calcification and PET tracer activity using Ga-68 DOTATATE PET/CT. Patients who underwent Ga-68 DOTATATE PET/CT imaging over a 12-month period for neuroendocrine investigation were included. Manual cardiac and aortic segmentations were performed by an experienced observer. Scans were randomly allocated in ratio 64:16:20 for training, validation and testing of the nnU-Net model. PET tracer uptake and calcium scoring were compared between segmentation methods and different observers. 116 patients (53.5% female) with a median age of 64.5 years (range 23–79) were included. There were strong, positive correlations between all segmentations (mostly r > 0.98). There were no significant differences between manual and AI segmentation of SUV_mean_ for global cardiac (mean ± SD 0.71 ± 0.22 vs. 0.71 ± 0.22; mean diff 0.001 ± 0.008, *p* > 0.05), ascending aorta (mean ± SD 0.44 ± 0.14 vs. 0.44 ± 0.14; mean diff 0.002 ± 0.01, *p* > 0.05), aortic arch (mean ± SD 0.44 ± 0.10 vs. 0.43 ± 0.10; mean diff 0.008 ± 0.16, *p* > 0.05) and descending aorta (mean ± SD < 0.001; 0.58 ± 0.12 vs. 0.57 ± 0.12; mean diff 0.01 ± 0.03, *p* > 0.05) contours. There was excellent agreement between the majority of manual and AI segmentation measures (r ≥ 0.80) and in all vascular contour calcium scores. Compared with the manual segmentation approach, the CNN required a significantly lower workflow time. AI segmentation of vascular contours using nnU-Net resulted in very similar measures of PET tracer uptake and vascular calcification when compared to an experienced observer and significantly reduced workflow time.

## Introduction

Atherosclerosis is a chronic inflammatory disease affecting medium and large-sized arteries that results from a diverse number of cellular and molecular processes. Tissue resident macrophages occupy a central role and are able to switch between pro- and anti-inflammatory functions as required [1]. Advances in vascular imaging continue to expand upon our understanding of the dysfunctional macrophage in cardiovascular disease. Several positron emission tomography (PET) tracers may facilitate a better understanding of atherosclerosis and guide translational opportunities for future therapies [2].

Deep learning or machine learning is a branch of artificial intelligence in which models are trained to mathematically extract features and draw inferences from inputs to address seemingly subjective problems. These models, which are designed to emulate human learning, build on prior experience and performance to refine their approach to tasks. These models are fed examples (trained) and taught to extract features in a hierarchical fashion, combining simpler concepts to learn more complex ones. Rather than deriving an analytical solution to a given problem—which may be arbitrarily complex, deep learning models develop a solution through iterative improvement of a previous model, updating the defined parameters to yield slightly improved outputs with respect to a performance metric (such as the Dice Similarity Coefficient) [3]. This cyclic process of predicting and updating can often be repeated until a satisfactory level of confidence is reached.

When the subject of these models is image-based, capturing the local context of data points becomes crucial to their interpretation—pixels are not observed in isolation but rather in clusters to derive meaning. Traditional neural networks vectorize their inputs, discarding all spatial significance of the data. To counteract this, the convolution operator which applies a matrix (kernel) of values to each pixel, taking a weighted average of it and all its surroundings was used. Each convolution kernel acts as a filter, and a network can use hundreds of these filters (each iteratively optimised) to identify pertinent features located at any point in the image. Networks utilising this tool are aptly given the name—Convolutional Neural Networks (CNNs).

A CNN model was developed using nnU-Net for automated segmentation of F-18 sodium fluoride (NaF) PET/CT cardiac imaging. The final model was decided following a thorough investigation of competing loss functions and model complexity. The final model used the DiceTopK10 and DiceTopK10CE loss functions and evaluated via overlap and distance-to-agreement measures [4]. Optimising the model further through loss function experimentation allowed for quickly attaining benchmarks quoted in the literature and exploring this avenue for clinical implementation. This research aims to re-train and investigate the performance of nnU-Net in the assessment of vascular contours on whole body historical Ga-68 dodecane tetraacetic acid-octreotate (DOTATATE) PET/CT studies.

## Methods

Scans performed on patients who underwent Ga-68 DOTATATE PET/CT imaging for detection or surveillance of neuroendocrine tumours over a 12 month period in 2012 were eligible. Imaging studies were performed on a Siemens Biograph mCT 64-slice PET/CT scanner at the Western Australian PET Service (Sir Charles Gairdner Hospital) per standard protocols with a 185 MBq tracer injection. Acquisition of PET and CT images was simultaneous and manual registration was not required. CT images were acquired for the purpose of anatomical localisation and were not cardiac gated. Patients under the age of 18 and those with non-diagnostic imaging quality were excluded from the study. There were no other exclusion or inclusion criteria. Ethics approval was obtained from East and North Metropolitan Human Research and Ethics Committees, which granted a waiver of the requirement for participant’s informed consent. Data analysis was carried out in accordance with National Health and Medical Research Council guidelines [5]. All scans were sequentially selected by date of imaging and randomly allocated with a ratio of 5:1 to a training group and a testing group.

A trained clinician experienced in PET/CT image analysis retrospectively analysed scans using a standardised workflow in MIM v7.0.6 (MIM Software Inc., Cleveland, OH) and performed manual segmentation of four vascular contours (the global cardiac silhouette, ascending aorta, aortic arch and descending thoracic aorta). Background activity was defined by contouring the right atrial blood pool. A semi-automated mask was applied with any non-vascular voxel of an SUV_max_ > 1.5 SUV excluded in 3 dimensions from all vascular contours by 6 mm to exclude spillover, largely from highly avid adjacent non-vascular structures such as the liver, spleen, and gastrointestinal tract. The clinician graded the impact of spillover on the contours on a four-point visual scale as either none, mild, major, or uninterpretable.

Manual segmentations were supplied by the clinician, each encoding four unique contour sequences: Ascending Aorta, Descending Aorta, Aortic Arch and Global Cardiac Silhouette. Studies were then divided in the ratio 64:16:20 for training, validation and testing of the nnU-Net model, respectively.

Image and contour files were first converted from DICOM to Nifti format, with nnU-Net handling the remaining pre-processing steps including cropping, augmenting, and resampling the images. Nifti formatted data provides much more manageable file sizes, convenient for moving, reading, loading and re-loading as performed regularly during analysis. Nifti is also required as default input to nnU-NET. The 3D nnU-NET model utilised the 3D image data. Images were cropped to the largest region containing non-zero values and resampled isotropically to the median voxel spacing utilising third order spline interpolation and nearest neighbour interpolation for image data and segmentation masks respectively. Augmentation was applied within the nnU-NET pipeline via random scaling, random rotation, gamma correction, mirroring and elastic deformation.

Patient CT data was supplied as the inputs, with the corresponding outputs benchmarked against the ground-truth binary segmentation maps from the clinician. A previous in-depth automated segmentation study conducted on a similar cardiac cohort (F-18 NaF PET/CT) offered a few useful insights into optimizing the modelling process. Firstly, PET data was omitted from the entire modelling pipeline due to marginal performance gains [4]. Secondly, the default nnU-Net loss function of Dice/Cross-Entropy Loss was replaced by a Dice/TopK10/Cross-Entropy loss function [6]. Incompatibilities between the two study’s image sizes meant the model had to be trained de novo without any transfer-learning.

Following training, predictions from the testing dataset were subsequently converted to DICOM format and reincorporated back into the MIM workflow. Statistical outputs from the automated method were compared against the corresponding statistics from the manually obtained contours.

To determine inter-observer agreement for manual segmentation a second, trained clinician with expertise in PET/CT image analysis manually contoured 10 studies by following the same workflow and employing identical methods, blinded to the output of the first clinician.

In vascular PET imaging, key parameters include SUV_max_ (maximum standardized uptake value), indicating the highest metabolic activity within a lesion; SUV_mean_ (mean standardized uptake value), providing an average metabolic measure across the region; and TBR_max_ (maximum target-to-background ratio), which enhances lesion specificity by comparing SUV_max_ to background tissue SUV_mean_. PET statistics were calculated on a per contour basis using standard methods. Calcium scoring was performed using the Agatston method on each contour [7]. Additional calculated statistics included contour volume, most diseased segment (MDS) SUV_max_, TBR_max_ and summed ‘thoracic aortic’ measures from individual ascending aorta, aortic arch and descending thoracic aorta contours.

### Statistical methods

Descriptive statistics included mean ± standard deviation (SD), median [25th to 75th percentile] and number (%) for Gaussian, non-Gaussian and categorical data. PET statistics were compared between manual and CNN segmentation methods using linear regression, paired sample t-tests, scatter plots and Bland–Altman plots [8]. Statistical analysis on studies was performed in SPSS v26 (IBM Corp, New York). Significance was set at a *p-*value below 0.05.

## Results

116 patient scans were included in the study. 96 scans were allocated to train the model and 20 were allocated to the testing groups. Of the 20 patient scans allocated to testing, the data from 2 patients was excluded due to gaps in the CT volume when downloaded from the server which resulted in errors in the original CT study formatting, preventing analysis by the nnU-Net model. The median age of the cohort was 64.5 years (range 23–79) with 61% male. Mean patient weight was 82.3 kg ± 14.76. Four patients of the test set (22%) had an Octreotate-positive neuroendocrine tumour at the time of the analysed study, compared to 20 of the training set (20.8%). Spillover from non-vascular structures was mild or absent for all scans (Table 1).

**Table 1:** Contour spillover characteristics

Spillover severity	Global cardiac silhouette	Ascending aorta	Aortic arch	Descending aorta
None	2 (11%)	16 (89%)	12 (67%)	3 (17%)
Mild	16 (89%)	2 (11%)	6 (33%)	15 (83%)
Major	0	0	0	0
Uninterpretable	0	0	0	0

**Fig. 1:**
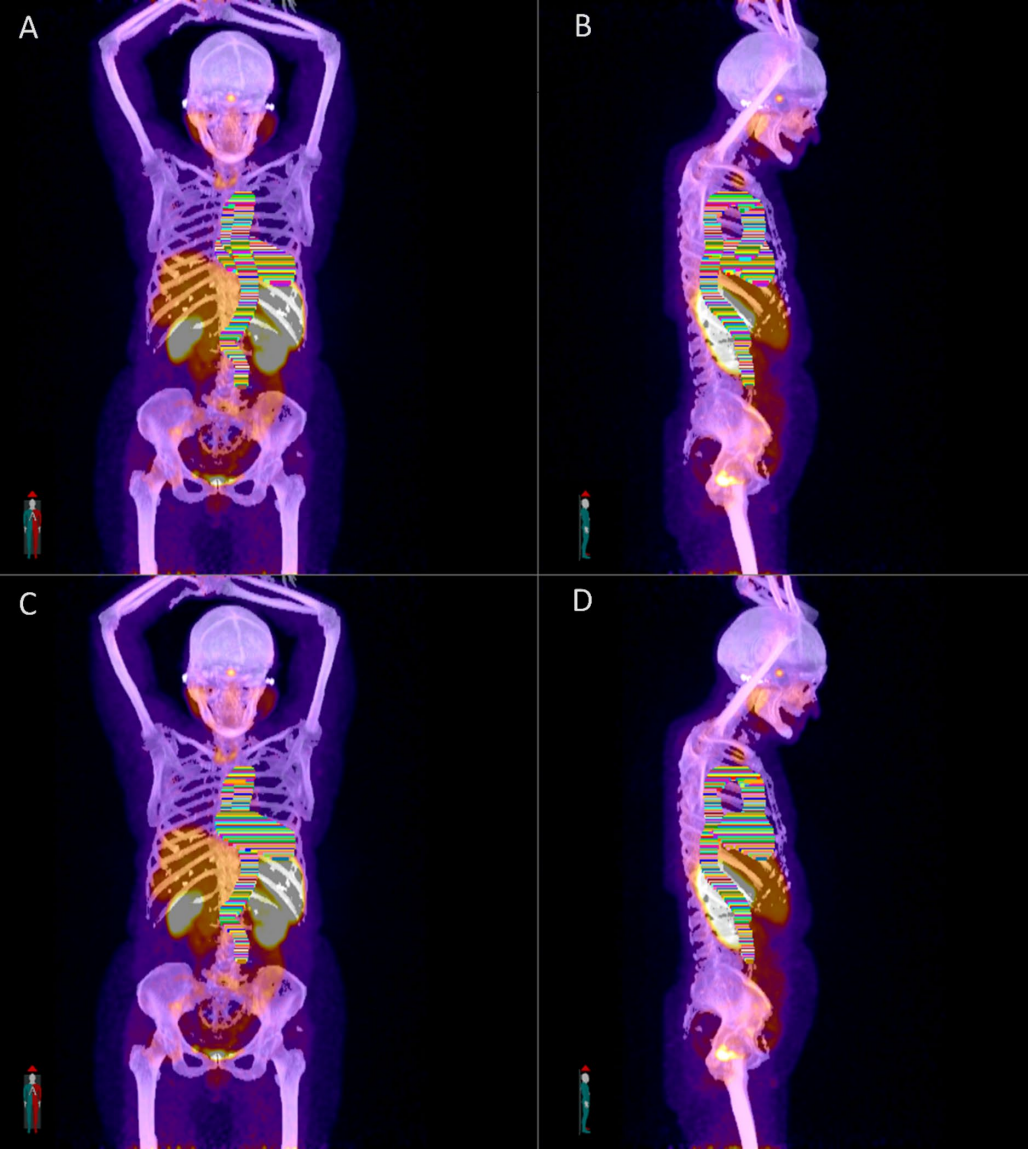
Segmentation of manual and AI contours on a representative patient using the described method. Panels **A** and **B** demonstrate manual segmentation regions of interest, **C** and **D** demonstrate AI segmentation regions of interest

The clinician imported the AI contours into MIM and performed study analysis per the established workflow. Mean analysis time for the clinician was significantly longer for manual vs AI segmentation: 1220 s ± 230.8 and 396 s ± 209.4; mean difference 825 s ± 71.7, *p*-value < 0.001. No contours required manual correction by the clinician. A representative example of the manual and AI contours is demonstrated in Fig. 1.

There was strong agreement between the two clinicians and no significant differences in SUV_mean_ for global cardiac (r = 1.00, *p*-value < 0.001: mean 0.67 ± 0.26 vs. 0.76 ± 0.36; mean diff 0.09 ± 0.22, *p*-value > 0.05), ascending aorta (r = 0.99, *p*-value < 0.001: mean 0.44 ± 0.14 vs 0.44 ± 0.14; mean diff 0.002 ± 0.01, *p*-value > 0.05), aortic arch (r = 0.98, *p*-value < 0.001: mean 0.45 ± 0.12 vs 0.45 ± 0.11; mean diff 0.004 ± 0.02, *p*-value > 0.05) and descending aorta (r = 0.99, *p*-value < 0.001: mean 0.57 ± 0.14 vs 0.57 ± 0.13; mean diff 0.002 ± 0.02, *p*-value > 0.05) contours. There was also strong agreement between the two clinicians in SUV_max_, TBR_max_ and calcification (see Table 2).

**Table 2:** Clinician agreements of SUV_mean_, SUV_max_, TBR_max_ and calcification

Measure	Contour	*r*-value	*p*-value	Mean ± SD	Mean difference ± SD	*p*-value
SUV_mean_	Global cardiac silhouette	1.000	< 0.001	0.67 ± 0.26	0.09 ± 0.22	NS
				0.76 ± 0.36		
	Ascending aorta	0.995	< 0.001	0.44 ± 0.14	0.002 ± 0.01	NS
				0.44 ± 0.14		
	Aortic arch	0.989	< 0.001	0.45 ± 0.12	0.004 ± 0.02	NS
				0.45 ± 0.11		
	Descending aorta	0.996	< 0.001	0.57 ± 0.14	0.002 ± 0.02	NS
				0.57 ± 0.13		
SUV_max_	Global cardiac silhouette	0.988	< 0.001	1.87 ± 0.57	0.01 ± 0.12	NS
				1.88 ± 0.64		
	Ascending aorta	0.954	< 0.001	1.06 ± 0.36	0.00 ± 0.11	NS
				1.07 ± 0.36		
	Aortic arch	0.859	0.001	1.09 ± 0.21	0.07 ± 0.01	NS
				1.16 ± 0.17		
	Descending aorta	0.932	< 0.001	1.28 ± 0.19	0.05 ± 0.07	NS
				1.23 ± 0.18		
TBR_max_	Global cardiac silhouette	0.856	< 0.001	4.74 ± 3.47	0.62 ± 2.02	NS
				4.13 ± 2.04		
	Ascending aorta	0.374	NS	2.56 ± 1.40	0.33 ± 1.36	NS
				2.22 ± 0.94		
	Aortic arch	0.926	< 0.001	2.84 ± 1.98	0.07 ± 0.75	NS
				2.77 ± 1.80		
	Descending aorta	0.855	< 0.001	3.35 ± 2.45	0.52 ± 1.39	NS
				2.83 ± 1.52		
Calcification	Global cardiac silhouette	0.987	< 0.001	64.6 ± 65.7	4.3 ± 11.0	NS
				60.3 ± 61.6		
	Thoracic aorta	0.993	< 0.001	1081.6 ± 2027.1	48.4 ± 240	NS
				1130.0 ± 2002.8		

*NS* not significant

**Fig. 2:**
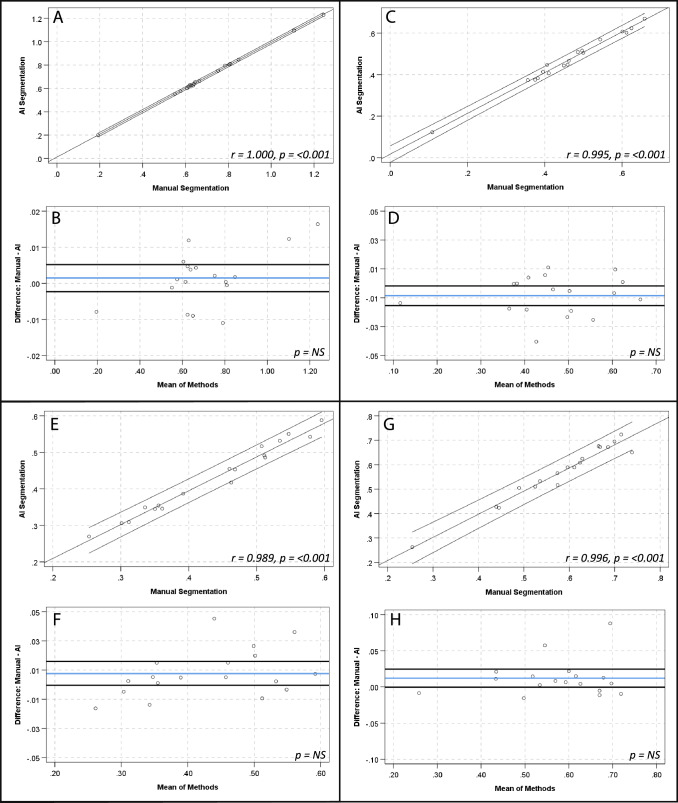
Scatter plots and Bland–Altman plots comparing manual and AI segmentation assessment of SUV_mean_ across contours; global cardiac silhouette (**A**, **B**), ascending aorta (**C**, **D**), aortic arch (**E**, **F**), descending aorta (**G**, **H**). Panels (**A**), (**C**), (**E**) and (**G**) demonstrate scatter plots with a linear line of best fit and 95% confidence intervals, with r and *p*-values inlaid on chart. Panels **B**, **D**, **F** and **H** demonstrate Bland–Altman plots with the mean difference (blue line), limits of agreement at ± 1.96*SD (black lines) and *p*-value inlaid on chart. *NS*  Not significant

**Fig. 3:**
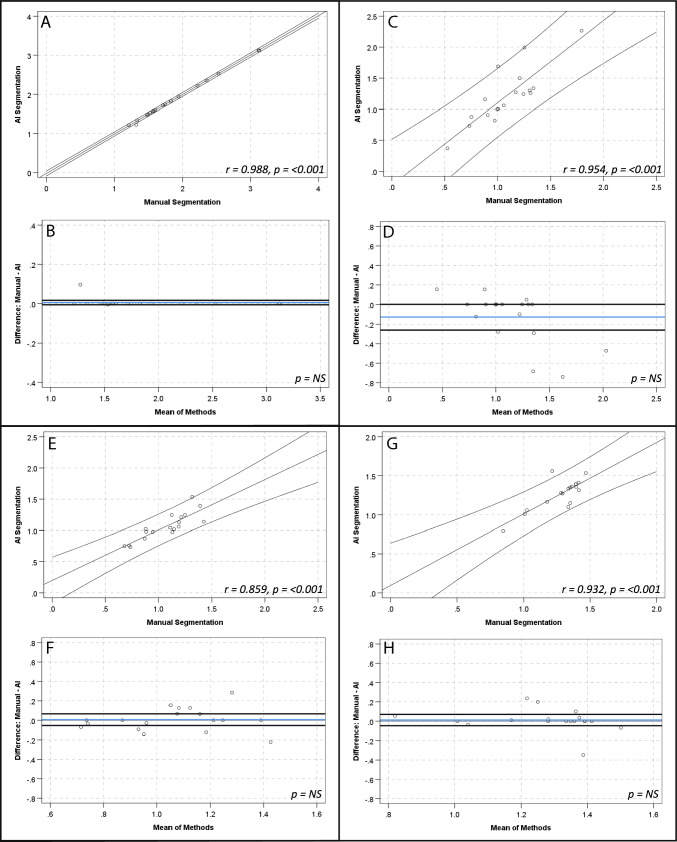
Scatter plots and Bland–Altman plots comparing manual and AI segmentation assessment of SUV_max_ across contours; global cardiac silhouette (**A**, **B**), ascending aorta (**C**, **D**), aortic arch (**E**, **F**), descending aorta (**G**, **H**). Panels (**A**), (**C**), (**E**) and (**G**) demonstrate scatter plots with a linear line of best fit and 95% confidence intervals, with r and *p*-values inlaid on chart. Panels (**B**), (**D**), (**F**) and (**H**) demonstrate Bland–Altman plots with the mean difference (blue line), limits of agreement at ± 1.96*SD (black lines) and *p*-value inlaid on chart. *NS*  Not significant

**Fig. 4:**
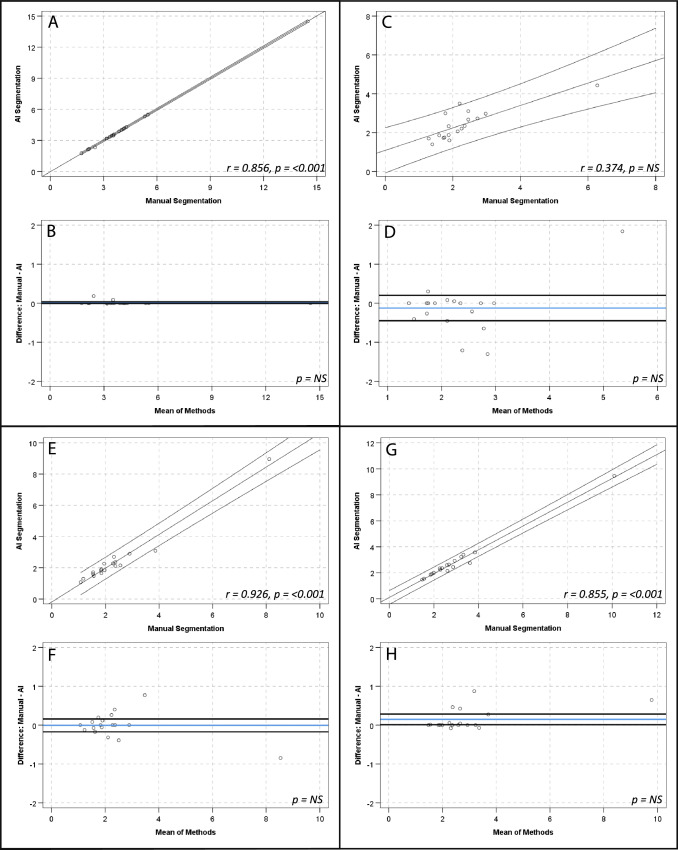
Scatter plots and Bland–Altman plots comparing manual and AI segmentation assessment of TBR_max_ across contours; global cardiac silhouette (**A**, **B**), ascending aorta (**C**, **D**), aortic arch (**E**, **F**), descending aorta (**G**, **H**). Panels (**A**), (**C**), (**E**) and (**G**) demonstrate scatter plots with a linear line of best fit and 95% confidence intervals, with r and *p*-values inlaid on chart. Panels (**B**), (**D**), (**F**) and (**H**) demonstrate Bland–Altman plots with the mean difference (blue line), limits of agreement at ± 1.96*SD (black lines) and *p*-value inlaid on chart. *NS*  Not significant

**Fig. 5:**
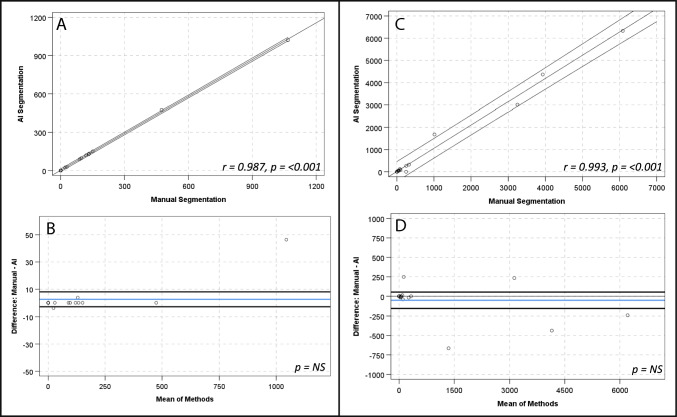
Scatter plots and Bland–Altman plots comparing manual and AI segmentation assessment of vascular calcification across contours; global cardiac silhouette (**A**, **B**), thoracic aorta (**C**, **D**). Panels (**A**) and (**C**) demonstrate scatter plots with a linear line of best fit and 95% confidence intervals, with r value and *p*-value inlaid on chart. Panels (**B**) and (**D**) demonstrate Bland–Altman plots with the mean difference (blue line), limits of agreement at ± 1.96*SD (black lines) and *p*-value inlaid on chart. *NS*  Not significant

**Fig. 6:**
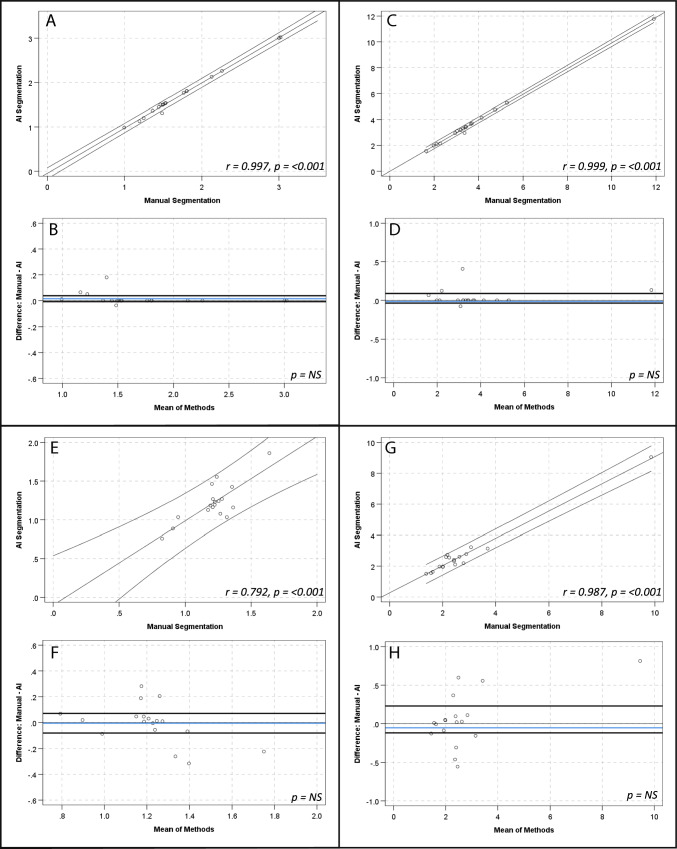
Scatter plots and Bland–Altman plots comparing manual and AI segmentation assessment of most diseased segment SUV_max_ and TBR_max_ across contours; most diseased segment SUV_max_ global cardiac silhouette (**A**, **B**), thoracic aorta (**C**, **D**), most diseased segment TBR_max_ global cardiac silhouette (**E**, **F**), thoracic aorta (**G**, **H**). Panels (**A**), (**C**), (**E**) and (**G**) demonstrate scatter plots with a linear line of best fit and 95% confidence intervals, with r and *p*-values inlaid on chart. Panels (**B**), (**D**), (**F**) and (**H**) demonstrate Bland–Altman plots with the mean difference (blue line), limits of agreement at ± 1.96*SD (black lines) and *p*-value inlaid on chart. *NS*  Not significant

There were strong, positive correlations and no significant differences between manual and AI segmentation of SUV_mean_ for global cardiac (*r* = 1.00, *p*-value < 0.001; mean 0.71 ± 0.22 vs 0.71 ± 0.22; mean diff 0.001 ± 0.008, *p*-value > 0.05), aortic arch (*r* = 0.98, *p*-value < 0.001; mean 0.44 ± 0.10 vs 0.43 ± 0.10; mean diff 0.008 ± 0.16, *p*-value > 0.05) and descending aorta (*r* = 0.97, *p*-value < 0.001; mean 0.58 ± 0.12 vs 0.57 ± 0.12; mean diff 0.01 ± 0.03, *p*-value > 0.05) contours. There was a strong, positive correlation for the ascending aorta SUV_mean_ (*r* = 0.99, *p*-value < 0.001; mean 0.46 ± 0.13 vs 0.47 ± 0.13; mean diff 0.009 ± 0.01, *p*-value 0.016) and a numerically small, but statistically significant difference of the means (0.009 ± 0.14, *p*-value 0.016) (see Fig. 2 and Table 3). The relationship between methods when assessing SUV_max_, TBR_max_ and calcification demonstrated similar results (see Table 3 and Figs. 3, 4 and 5, respectively). Analysis of global cardiac and aortic contour most diseased segment SUV_max_ and TBR_max_ demonstrated strong, positive correlations and no significant differences between methods for all measures (see Table 4 and Fig. 6).

**Table 3:** Comparison of SUV_mean_, SUV_max_, TBR_max_ and vascular calcification assessment by manual and AI segmentation

Measure	Contour	*r*-value	*p*-value	Mean ± SD	Mean difference ± SD	*p*-value
SUV_mean_	Global cardiac silhouette	1.000	< 0.001	0.71 ± 0.22	0.001 ± 0.007	NS
	Global cardiac silhouette (AI)			0.71 ± 0.22		
	Ascending aorta	0.994	< 0.001	0.46 ± 0.13	0.01 ± 0.01	0.016
	Ascending aorta (AI)			0.47 ± 0.13		
	Aortic arch	0.989	< 0.001	0.44 ± 0.10	0.01 ± 0.02	NS
	Aortic arch (AI)			0.43 ± 0.10		
	Descending aorta	0.978	< 0.001	0.58 ± 0.12	0.1 ± 0.03	NS
	Descending aorta (AI)			0.57 ± 0.12		
SUV_max_	Global cardiac silhouette	0.999	< 0.001	1.87 ± 0.58	0.01 ± 0.02	NS
	Global cardiac silhouette (AI)			1.87 ± 0.58		
	Ascending aorta	0.839	< 0.001	1.08 ± 0.29	0.13 ± 0.26	NS
	Ascending aorta (AI)			1.21 ± 0.45		
	Aortic arch	0.855	< 0.001	1.07 ± 0.23	0.01 ± 0.12	NS
	Aortic arch (AI)			1.06 ± 0.22		
	Descending aorta	0.795	< 0.001	1.28 ± 0.17	0.01 ± 0.12	NS
	Descending aorta (AI)			1.27 ± 0.19		
TBR_max_	Global cardiac silhouette	1.000	< 0.001	4.14 ± 2.77	0.01 ± 0.05	NS
	Global cardiac silhouette (AI)			4.12 ± 2.78		
	Ascending aorta	0.805	< 0.001	2.28 ± 1.10	0.12 ± 0.33	NS
	Ascending aorta (AI)			2.40 ± 0.78		
	Aortic arch	0.984	< 0.001	2.39 ± 1.57	0.01 ± 0.33	NS
	Aortic arch (AI)			2.40 ± 1.72		
	Descending aorta	0.992	< 0.001	2.97 ± 1.90	0.15 ± 0.27	0.036
	Descending aorta (AI)			2.82 ± 1.76		
Calcification	Global cardiac silhouette	1.000	< 0.001	261.0 ± 61.5	2.6 ± 11.0	NS
	Global cardiac silhouette (AI)			126.0 ± 251.2		
	Thoracic aorta	0.994	< 0.001	858.1 ± 1732.6	51.9 ± 211.7	NS
	Thoracic aorta (AI)			910.0 1 ± 1819.5		

*NS* Not significant (*p*-value > 0.05)

**Table 4:** Comparison of most diseased segment SUV_max_ and TBR_max_ assessment by manual and AI segmentation

Measure	Contour	*r*-value	*p*-value	Mean ± SD	Mean difference ± SD	*p*-value
MDS SUV_max_	Global cardiac silhouette	0.997	< 0.001	1.72 ± 0.56	0.02 ± 0.05	NS
	Global cardiac silhouette (AI)			1.71 ± 0.57		
	Thoracic aorta	0.792	< 0.001	1.21 ± 0.18	0.006 ± 0.15	NS
	Thoracic aorta (AI)			1.22 ± 0.25		
MDS TBR_max_	Global cardiac silhouette	0.999	< 0.001	3.74 ± 2.23	0.04 ± 0.10	NS
	Global cardiac silhouette (AI)			3.71 ± 2.22		
	Thoracic aorta	0.987	< 0.001	2.74 ± 1.87	0.05 ± 0.35	NS
	Thoracic aorta (AI)			2.70 ± 1.66		

*MDS* Most diseased segment*, NS* Not significant (*p*-value > 0.05)

**Fig. 7:**
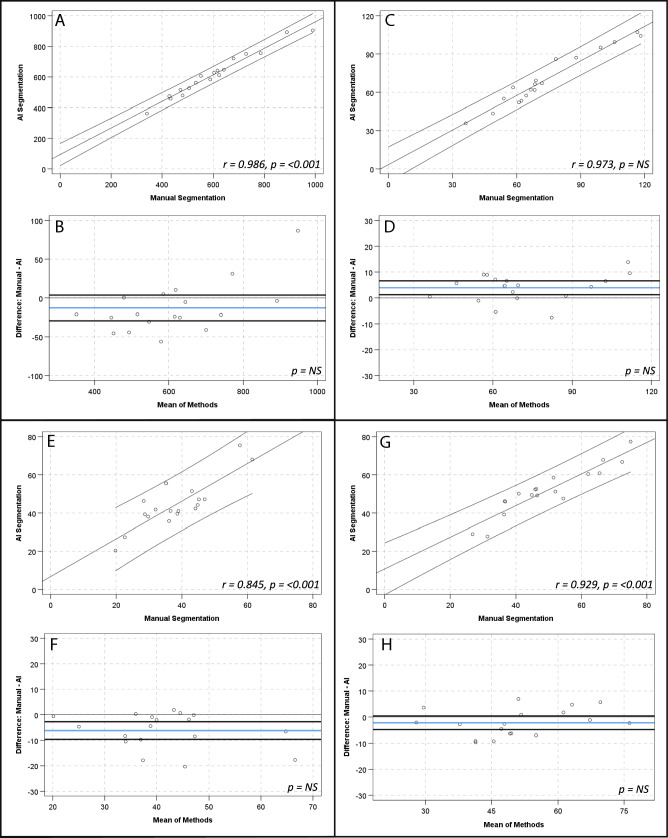
Scatter plots and Bland–Altman plots comparing manual and AI segmentation assessment of contour volume across contours; global cardiac silhouette (**A**, **B**), ascending aorta (**C**, **D**), aortic arch (**E**, **F**), descending aorta (**G**, **H**). Panels (**A**) and (**C**) demonstrate scatter plots with a linear line of best fit and 95% confidence intervals, with r and *p*-values inlaid on chart. Panels (**B**) and (**D**) demonstrate Bland–Altman plots with the mean difference (blue line), limits of agreement at ± 1.96*SD (black lines) and *p*-value inlaid on chart. *NS*  Not significant

There were strong, positive correlations between manual and AI segmentation of contour volumes for all contours and numerically small but statistically significant differences in ascending aorta and aortic arch volumes (mean diffs 3.9 ± 5.4 ml and 6.2 ± 6.8 ml, respectively, *p*-values < 0.01) (see Table 5 and Fig. 7).

**Table 5:** Comparison of contour volume (ml) by manual and AI segmentation

Contour	*r*-value	*p*-value	Mean ± SD	Mean difference ± SD	*p*-value
Global cardiac silhouette	0.986	< 0.001	604.6 ± 165.9	12.9 ± 33.1	NS
Global cardiac silhouette (AI)			617.5 ± 145.7		
Ascending aorta	0.973	< 0.001	72.2 ± 22.8	3.9 ± 5.4	0.007
Ascending aorta (AI)			70.3 ± 21.2		
Aortic arch	0.845	< 0.001	38.3 ± 11.0	6.2 ± 6.8	0.001
Aortic arch (AI)			44.6 ± 12.8		
Descending aorta	0.929	< 0.001	49.6 ± 14.1	2.2 ± 5.3	NS
Descending aorta (AI)			51.8 ± 12.6		

*NS* Not significant (*p*-value > 0.05)

## Discussion

The present study provides evidence for the substantial benefits of using nnU-Net, a publicly available CNN [9]. A robust positive correlation was demonstrated between manual segmentation and AI segmentation, with limited disparities in the methodologies. While statistically significant differences were observed, they were quantitatively trivial and arose primarily from slight variations in contour definition, as in the case of calcification at the interface of two contours, such as the ascending aorta and aortic arch, leading to contrasting calcium scores based on the contour delineation.

This study has several limitations. The use of masking techniques was necessary due to the pattern of Ga-68 DOTATATE tracer uptake, and manual segmentation remains inherently variable despite efforts to standardize anatomical and non-anatomical contour boundaries. Challenges arise when attempting to differentiate the inner and outer aortic walls due to the thin nature of the vessel lining, potential partial volume effects (leading to potential inaccuracies in quantifying tracer uptake and inflammation) and the use of a CT performed for the purposes of attenuation correction. These limitations may result in small variations in predicted contours of complex structures, leading to significant differences in contouring (as was observed with outliers in aortic and most diseased segment contours) and may have implications for diagnostic, therapeutic planning, and research purposes. Further investigation is necessary to determine the optimal size of the training dataset for clinical use, establish the acceptable level of variability, and clarify the role of the clinician in advanced segmentation (especially in vascular territories), considering the presence of intravascular stents, surgical material, and variant or rare anatomy.

Small vascular lesions are prone to partial volume artifact, low target-to-background ratios, and the proximity of the blood pool which represent challenges to tracer development and image interpretation, constraints gradually being addressed by technological advances including total body PET, new image reconstruction and motion correction techniques and hybrid tracer imaging using nanoparticles [10–12].

Results of the present work align with previous research reporting significant improvements in both analysis time and contour accuracy with the use of a CNN compared to manual segmentation [13]. The performance of nnU-Net (in particular) has been extensively evaluated for non-vascular segmentation and has consistently demonstrated strong performance across a wide range of applications, including neuroradiology, cardiology, musculoskeletal injury and oncology [14–17]. Manual segmentation is known to be a labour-intensive process and the present study demonstrates the significant decrease in workflow time that can be achieved. Deep learning based automated segmentation has the potential to improve efficiency and reproducibility to a clinically acceptable standard equal to, or even greater, than can be attained by trained clinicians [18, 19]. Implementing such techniques in the clinical workflow would both streamline and improve quality of diagnosis, utilisation of senior clinician time and improve the access and affordability of non-invasive functional imaging. This reduction in time represents an improvement in the utilization of a skilled workforce, increasing efficiency, and enhancing productivity. Furthermore, these findings translate to cost savings for healthcare services. Importantly, the performance of the model was not significantly impaired across a diverse patient population, including those with and without severe vascular calcification.

One of the most cited, and foundational CNNs used for deep-learning experimentation in medical imaging is the U-Net architecture [20]. The network is comprised of a contracting and expanding path, symmetric in their use of down- and up-sampling operators, giving the model it’s identifiable "U" shape. U-Net and its variants have demonstrated high accuracy in segmenting biomedical images and wide applicability [21–27]. Rapid advancement across a range of both open-source and proprietary AI models has led to advances in CT-FFR, improvements in cardiovascular event prediction by nuclear perfusion imaging, and myocardial tissue characterisation on cardiac MRI [28, 29]. However, replicating published benchmarks requires careful modification of model configurations and training schemes, catering to the characteristics of choice datasets. This is especially prevalent in three-dimensional biomedical imaging problem domains, where imaging modality, anisotropic voxel spacing, and imaging dimensionality may vary dramatically between facilities. The high dimensionality of model hyperparameter configuration, coupled with the limited supply of training and validation data, thus often leads to models failing to live up to their promised performance when evaluated on similar, but unseen problem domains.

nnU-Net aids in navigating this complex parameter domain by handling all the pre-processing, training, and inference-making in the prediction pipeline. To achieve this, design choices are based on the data itself considering such factors as voxel-spacing, image dimensions and class ratios. Streamlining this greatly reduces the hyperparameter tuning domain, allowing the user to quickly generate literature-comparable results and build from there.

The use of AI in medical imaging represents a major stride forward in the advancement of healthcare. The present study serves as a demonstration of the capabilities of AI and its ability to provide more efficient and accurate results than traditional methods. The findings of this study have significant implications for the field of medical imaging and provide compelling evidence for the continued investment in research and development of AI in the healthcare sector. With the continued advancement of AI technologies, the ability to analyse complex medical images and generate data from large datasets will only continue to improve. This, in turn could streamline clinical trial conduct, provide a platform for personalised medicine and ultimately, improve health outcomes for patients. New knowledge gained from this study includes demonstration of strong, positive agreement in clinical measures of vascular tracer uptake and calcification beyond contour Dice coefficients, alongside a significant reduction in clinician workload.

## Conclusion

Automated segmentation of the global cardiac silhouette and aortic contours using the nnU-Net CNN demonstrated excellent performance when compared to a trained clinician in the assessment of SUV_mean_ and other measures, was associated with a significant reduction in workflow completion time and did not require manual corrections.

## Abbreviations

CNN: Convolutional neural network
CT-FFR: Computed tomography fractional flow reserve
MDS: Most diseased segment
NNU-Net: No-new-U-net
SUV: Standardised uptake values
TBR: Target-to-background ratio
